# Low-Frequency Fluctuations of the Resting Brain: High Magnitude Does Not Equal High Reliability

**DOI:** 10.1371/journal.pone.0128117

**Published:** 2015-06-08

**Authors:** Dewang Mao, Zhongxiang Ding, Wenbin Jia, Wei Liao, Xun Li, Huiyuan Huang, Jianhua Yuan, Yu-Feng Zang, Han Zhang

**Affiliations:** 1 Zhejiang Provincial People’s Hospital, Hangzhou, Zhejiang Province, P. R. China; 2 Center for Cognition and Brain Disorders, Hangzhou Normal University, Hangzhou, Zhejiang Province, P. R. China; 3 Zhejiang Key Laboratory for Research in Assessment of Cognitive Impairments, Hangzhou, Zhejiang Province, P. R. China; University Of Cambridge, UNITED KINGDOM

## Abstract

The amplitude of low-frequency fluctuation (ALFF) measures low-frequency oscillations of the blood-oxygen-level-dependent signal, characterizing local spontaneous activity during the resting state. ALFF is a commonly used measure for resting-state functional magnetic resonance imaging (rs-fMRI) in numerous basic and clinical neuroscience studies. Using a test-retest rs-fMRI dataset consisting of 21 healthy subjects and three repetitive scans, we found that several key brain regions with high ALFF intensities (or magnitude) had poor reliability. Such regions included the posterior cingulate cortex, the medial prefrontal cortex in the default mode network, parts of the right and left thalami, and the primary visual and motor cortices. The above finding was robust with regard to different sample sizes (number of subjects), different scanning parameters (repetition time) and variations of test-retest intervals (i.e., intra-scan, intra-session, and inter-session reliability), as well as with different scanners. Moreover, the qualitative, map-wise results were validated further with a region-of-interest-based quantitative analysis using “canonical” coordinates as reported previously. Therefore, we suggest that the reliability assessments be incorporated in future ALFF studies, especially for the brain regions with a large ALFF magnitude as listed in our paper. Splitting single data into several segments and assessing within-scan “test-retest” reliability is an acceptable alternative if no “real” test-retest datasets are available. Such evaluations might become more necessary if the data are collected with clinical scanners whose performance is not as good as those that are used for scientific research purposes and are better maintained because the lower signal-to-noise ratio may further dampen ALFF reliability.

## Introduction

Low-frequency fluctuation of the blood-oxygen-level-dependent (BOLD) signal is an important characteristic of the resting-state brain [1–5]. It reflects the strength of brain activity. One of the commonly used and easiest approaches for the investigation of this characteristic is analysis of the amplitude of low-frequency fluctuation (ALFF) [6–8].

Proposed by Zang et al. [7], ALFF has been used to measure local spontaneous activity using resting-state fMRI (rs-fMRI) and has been applied in investigations of various neurological and psychological diseases [9–19] and of normal cognitive functions [20–22]. In those studies, a high ALFF value (or, to maintain consistency, “ALFF magnitude”, will be used from here on) was consistently found in the so-called “default model network” (DMN), one of the most widely investigated brain networks [1,6,23–27]. This indicates a high baseline metabolism level in those areas. Alterations in the ALFF magnitude are often found in those areas and are sometimes proposed as biomarkers of diseases [10,16,28]. Therefore, whether the high ALFF magnitude in the DMN areas is biologically meaningful or not has become an important scientific issue.

Zuo et al. [29] conducted a systematic investigation of the test-retest reliability of the ALFF map, and the results indicated that the voxel-wise ALFF is largely reliable within scanning sessions and across sessions. In their study, intra-class correlation (ICC) was also calculated in a region-of-interest (ROI)-wise manner, and the ALFF magnitude in DMN areas was found to be consistently high across scans and subjects. Zuo et al. [29] mainly focused on the regions with high reliability (i.e., the peak coordinates of ICC maps) and on the difference in reliability between the ALFF and fractional ALFF (fALFF, a derivative of ALFF, which calculates the normalized ALFF by dividing the whole frequency amplitude [8]), as well as the ranking consistency of the ALFF magnitude among all the ROIs across scans and subjects. However, it remains unclear whether the voxels with high ALFF magnitude (an important indicator of resting state-related functions) also have high ALFF reliability. Therefore, the ALFF map deserves a dedicated study with a voxel-wise assessment comparing ALFF magnitude and reliability. This is essential before the ALFF, as a voxel-wise measurement, can be considered a biomarker in clinical studies.

In this study, we aimed to answer this question and to provide guidelines for how to report ALFF results with regard to the relationship between ALFF magnitude and reliability.

## Materials and Methods

### Subject information

Twenty-one healthy subjects (11 males, 10 females; age 23–49 years [26.5 ± 5.9]) were enrolled from local communities. All subjects were right-handed, native Chinese speakers. None of them had a history of neuropsychological disease or language, hearing or visual impairments. This study was approved by the ethics committee of Zhejiang Provincial People’s Hospital (approval number: 2013KY064). All subjects signed a written informed consent form before the scans were conducted.

### Scanning parameters

In the first session, two rs-fMRI scans (denoted as “RS1” and “RS2”) were acquired for all subjects using a Siemens 3.0T Trio MR scanner in the Zhejiang Provincial People’s Hospital with a circular polarized (CP) array head coil, which produces highly homogeneous functional images, with no parallel imaging. During the rs-fMRI scans, the subjects were asked to remain motionless, close their eyes, and relax. The RS1 and the RS2 scans were separated with an inter-scan interval of 5 min, during which the subjects were asked to lie motionless in the scanner. After two weeks, ten (5 males, 5 females; age 23–34 years [25.6 ± 3.86]) of the 21 subjects underwent a third rs-fMRI scan (RS3) using the same scanner. The other subjects, because of their absence when performing the third scan, were only scanned twice. The scanning parameters were as follows: repetition time (TR), 2 s; echo time, 30 ms; flip angle, 90°; slice thickness, 3.5 mm; slice interval, 0.7 mm; matrix size, 64 × 64; voxel size, 3.4 × 3.4 × 4.2 mm^3^; field of view, 220 × 220 mm; and slice number, 31 (interleaved scanning order). The total scanning time for each rs-fMRI scan was 8 min and produced 240 volumes.

### Data preprocessing

The data from the RS1, RS2, and RS3 scans were preprocessed using Matlab (MathWorks, Inc., Natick, MA), Statistical Parametric Mapping toolbox (SPM8, http://www.fil.ion.ucl.ac.uk/spm), REST version 1.8 [30] and DPARSFA version 2.2 [31] (http://www.restfmri.net). The first five images of each scan were discarded to allow stabilization of the blood-oxygen-level-dependent (BOLD) signal. The remaining functional data were slice-timing corrected, head motion corrected, and then subjected to spatial normalization using the EPI template to register the individual rs-fMRI data to the Montreal Neurological Institute (MNI) space. The data were further re-sampled to a resolution of 3 × 3 × 3 mm^3^ and spatially smoothed using an 8 mm full-width half-maximum Gaussian kernel. Subjects with head motions larger than 2 mm or 2° were excluded from further analyses. No subject was excluded according to this criterion. In addition, the frame-wise displacement (FD) for characterizing “micro” head movements from one time point to the next was also calculated for each subject because such movements could introduce systematic artifactual temporal correlations among voxels and thus may affect reliability assessment. The mean and variability of the number of large (FD > 0.2) FD time points were calculated for each rs-fMRI scan and compared between scans (e.g., RS1 vs. RS2, RS2 vs. RS3) to see if greater micro-head motion could induce lower reliability. Finally, all of the data were detrended to remove BOLD signal drift. The preprocessed data entered the following analyses. In addition, to address the potential effect of nuisance signals such as the head motion parameters, the white matter signal and the cerebrospinal fluid signal on ALFF estimation [29], we further regressed out these nuisance signals from our data. The results with and without further nuisance signal regression were quite similar (S1 Fig).

### ALFF calculation and statistical analyses

ALFF was calculated at a frequency band of 0.01–0.08 Hz according to Zang et al. [7] for each subject and for each run. The ALFF maps were then transformed to *z* maps (zALFF). For RS1 and RS2, group-level ALFF maps were calculated using the one-sample *t* test on the zALFF maps (p < 0.00001, cluster size > 40, uncorrected). For RS3, a group-level ALFF map was obtained (p < 0.001, cluster size > 40, uncorrected). Of note, the group analyses were performed on the global mean-subtracted and global variation-divided rather than the original “raw” individual ALFF images to avoid the potential confounding of subject-specific whole-brain overall ALFF on the reliability estimate. To accomplish this, for each subject, the original “raw” ALFF value in each voxel was subtracted using this subject’s averaged ALFF value across all voxels in a whole brain mask and was then divided by the standard deviation of ALFF values across all voxels in the whole brain mask. The three *t* maps (for RS1, RS2 and RS3) were generated for visual presentation and comparison.

### Map-wise ALFF magnitude/reliability assessment

To calculate overall map-wise intra- and inter-session reliability at the group level, the averaged zALFF map across subjects within each run was generated, and the spatial similarity between each pair among the averaged RS1, RS2 and RS3 zALFF maps was assessed using a Pearson correlation.

### Voxel-wise ALFF magnitude/reliability assessment (21 subjects, intra-session: RS1/2)

To carry out a voxel-wise comparison between the ALFF magnitude and reliability, we calculated the average zALFF map for all the subjects. For each of the 21 subjects, the zALFF maps from the RS1 and the RS2 scans were first averaged; then, the resultant mean zALFF maps were further averaged for all the 21 subjects, forming a group zALFF magnitude map. To assess the ALFF reliability for RS1 and RS2, a voxel-wise intra-session test-retest reliability of zALFF was calculated using the intraclass correlation coefficient (ICC), thereby forming a reliability map (intra-session reliability between measurements RS1 and RS2). The ICC calculation was based on a one-way analysis of variance model with random subject effects, where the total sum of the variance squares was separated into between-subject (MS_b_) and within-subject (MS_w_, i.e., residual error) sum of squares (see Eq 1, where *k* is the number of repeated observations per subject [32], and in this case, *k* = 2).

$$\text{ICC}=\;\frac{{\text{MS}}_{\text{b}}-{\text{MS}}_{\text{w}}}{{\text{MS}}_{\text{b}}+\;\left(\text{k}-1\right){\text{MS}}_{\text{w}}}$$ (1)

Voxels with ICC values larger than 0.5 were considered to be reliable [29,33]. The ALFF magnitude map and the ALFF reliability map were thresholded (averaged *z* > 1.5, ICC > 0.5) and compared.

### Voxel-wise ALFF magnitude/reliability assessment (10 subjects, inter-session: RS1/2/3)

For the 10 subjects who underwent all three scans, analyses similar to those above were conducted to comprehensively assess the ALFF test-retest reliability, with inter-session reliability taken into account. In this case, the ALFF magnitude map was calculated by averaging the zALFF maps across the three scans and then across the 10 subjects. The ALFF reliability map was calculated using ICC, according to Eq 1, with *k* = 3 (RS1, RS2 and RS3). The ALFF magnitude and reliability maps were thresholded (averaged *z* > 1.5, ICC > 0.5) and compared.

### ROI-wise ALFF magnitude/reliability assessment

**Fig 1:**
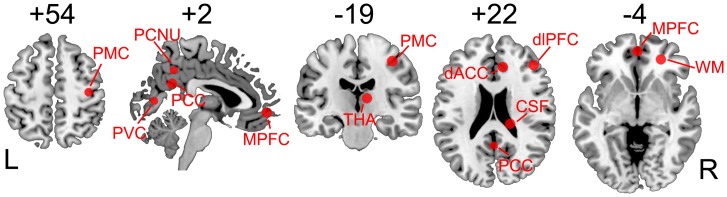
ROIs selected to compare ALFF magnitude with ALFF test-retest reliability. PMC: primary motor cortex, PVC: primary visual cortex, PCC: posterior cingulate cortex, MPFC: medial prefrontal cortex, PCNU: precuneus, THA: thalamus, dACC: dorsal anterior cingulate cortex, dlPFC: dorsolateral prefrontal cortex, CSF: cerebrospinal fluid, WM: white matter. Note that all the ROIs were either in the midline structure or on the right side. The underlying structural brain image is the CH2 template in the Montreal Neurological Institute space.

To emphasize our voxel-wise finding and to clearly demonstrate the relationship between the magnitude and the test-retest reliability of the ALFF, we performed a ROI analysis, extracting and comparing the averaged zALFF values across runs and subjects with the ICC values in several ROIs. The ROIs were selected from the key nodes of our interested resting-state networks from the rs-fMRI literature or were based on anatomical landmarks (for details of ROI definitions, please see Fig 1 and Table 1). Of note, this was not a circular analysis because our aim here was not to report any new findings but to evaluate the result from another perspective. To prevent potential bias, the ROIs were selected by another colleague who was blinded to the map- and voxel-wise results. Ten ROIs in the DMN, salience network (including the dorsal anterior cingulate cortex [dACC] and the dorsolateral prefrontal cortex [dlPFC]), thalamus, primary motor cortex (PMC) and primary visual cortex (PVC), as well as non-grey matter areas (white matter [WM] and cerebrospinal fluid in the lateral ventricles [CSF]), were defined according to previous studies [28,34,35] or anatomical landmarks and existing atlases, including Automated Anatomical Labeling (AAL) [36] and Brodmann’s areas (BA). The ROI-wise assessment was performed both for RS1/2 and for RS1/2/3.

**Table 1:** Regions-of-interest (ROIs) selected in this study.

	MNI coordinates	Reference
**PCC**	(0, -53, 26)	Hedden et al., 2009
**MPFC**	(0, 52, -6)	Hedden et al., 2009
**PCNU**	(-2, -51, 41)	Lynch et al., 2013
**dACC**	(10, 34, 24)	Seeley et al., 2007
**dlPFC**	(44, 36, 20)	Seeley et al., 2007
**THA**	(10, -21, 10)	Centering in the thalamus (AAL)
**PMC**	(38, -19, 51)	Landmark of hand area in the precentral gyrus
**PVC**	(6, -73, 7)	Centering in the calcarine (AAL) and BA17
**WM**	(26, 43, -6)	Anatomical information
**CSF**	(18, -29, 24)	Anatomical information

PCC: posterior cingulate cortex, MPFC: medial prefrontal cortex, PCNU: precuneus, dACC: dorsal anterior cingulate cortex, dlPFC: dorsolateral prefrontal cortex, THA: thalamus, PMC: primary motor cortex, PVC: primary visual cortex, WM: white matter, CSF: cerebrospinal fluid, MNI: Montreal Neurological Institute, AAL: Automated Anatomical Labeling, BA: Brodmann’s area. Note that all the ROIs were either in the midline structures or on the right side.

### ALFF magnitude/reliability assessment in various frequency bands

As Zuo et al. [29] found a different ALFF reliability character when it was calculated in different frequency bands, we also attempted to determine if a large ALFF magnitude corresponding to low reliability is a universal phenomenon across different frequency bands. Therefore, in addition to the conventional low-frequency band (0.01–0.08 Hz), we also calculated the ALFF in different frequency bands: slow-5 (0.01–0.027 Hz), slow-4 (0.027–0.073 Hz), slow-3 (0.073–0.198 Hz) and slow-2 (0.198–0.25 Hz) [29]. The inter-session ICC was calculated with each frequency band using the RS1, RS2 and RS3 scans from 10 subjects. Comparisons were performed among the four ICC images and the four group-averaged zALFF images obtained from four frequency bands. The ICC values and the mean zALFF values in each of the 10 ROIs specified above were also plotted against different frequency bands.

### ALFF magnitude/reliability estimates against different test-retest intervals

As the ALFF was calculated typically between 0.01 and 0.08 Hz, it can be calculated based on a minimum scanning length of approximately 100 s. To systematically investigate the relationship between ALFF magnitude and reliability as well as such a relationship against different test-retest intervals, we divided each of the 8 min rs-fMRI data sets into three segments (each consisting of 156 s data). This allowed us to assess the reliability of the ALFF with variations of test-retest intervals, from “intra-scan”, to “intra-session” and “inter-session” reliability, which was helpful for assessing which factor(s) might contribute to the ALFF instability. The analysis procedure was as follows: (1) calculate 10 (subjects) × 3 (rs-fMRI scans) × 3 (data segments) = 90 zALFF images; (2) calculate ICC across the 3 data segments and repeat it for every rs-fMRI scan; (3) average the three ICC maps generated in step 2 across the three rs-fMRI scans (RS1/2/3), generating an “intra-scan” ALFF reliability map; (4) calculate “intra-session” ICC using all of the 3 × 2 = 6 data segments from rs-fMRI scans RS1 and RS2; and (5) calculate the “inter-session” ICC using all of the 3 × 3 = 9 data segments from rs-fMRI scans RS1, RS2 and RS3. Comparisons were conducted among the “intra-scan”, “intra-session” and “inter-session” ICC images in both voxel- and ROI-wise manners.

### Validation of ALFF magnitude/reliability with an independent dataset

We also applied the same process to an independent dataset to further validate whether a relationship between ALFF magnitude and reliability can be consistently obtained. A publicly accessible dataset, the “initial test-retest dataset in the project of the enhanced Nathan Kline Institute-Rockland Sample (NKI-RS)”, was used for this purpose. We used only a part of the dataset that had been obtained during resting-state with a standard EPI sequence (repetition time, 2500 ms, 3-mm isotropic voxels, total scanning time, 5 min). For more details on these data, please visit http://fcon_1000.projects.nitrc.org/indi/pro/eNKI_RS_TRT/FrontPage.html. Twenty-four subjects were each scanned twice at an interval of approximately 1 week. Due to excessive head motions according to the same criteria, 2 subjects were excluded, thus 22 subjects (16 males, 6 females, age 34.4 ± 12.5 years) were remained. All of the data processing procedures were maintained the same as those used for our data. An averaged zALFF map across sessions and subjects was obtained, and was compared with the zALFF ICC map that was calculated using this dataset.

### Reliability of ALFF based on short-TR rs-fMRI data

The Nyquist-Shannon sampling theorem prevents from an accurate estimation of the ALFF from the rs-fMRI data with a TR longer than 1/(noise frequency × 2). The present data had TR = 2s, likely causing a high-frequency artifactual signal (i.e., cardiac pulse signal) to blend into the low-frequency band where the ALFF was calculated. In this case, the ALFF instability among scans could be due to these artificial sources. To rule out an inadequate temporal sampling issue, we utilized another independent eyes-closed rs-fMRI dataset with short TR (TR = 400 ms, TE = 15 ms, Flip angle = 30 degree, Slice number = 13, Slice thickness = 6 mm, scanning time = 8 min) and applied a similar analysis to it. For all 46 subjects (age 22–32 years, 23 females) in this dataset, the rs-fMRI data were split into three segments, each of which consisted of 156 s data (390 volumes, i.e., the minimum data length allowing an ALFF calculation between 0.01 and 0.08 Hz). An intra-scan ICC map was calculated across the 3 segments. For more details on the data and processing information, please see S1 Text. Because the ICC was calculated in an intra-scan manner and because thicker slices will increase the signal-to-noise ratio (SNR) and thus increase reliability, we set a higher threshold (i.e., ICC > 0.6) to compare the short-TR results fairly with the results from a typical TR (i.e., 2s).

## Results

### Head motion contribution to ALFF reliability

The percentage of the FD values [37] larger than 0.2 for the RS1, RS2 and RS3 scans were 7.73±3.74%, 7.53±3.95% and 6.03±3.25%, respectively. The difference in the value between RS1 and RS2 (intra-session head motion difference) for the 21 subjects was 0.19±1.98%, and that between RS1 (or RS2) and RS3 (inter-session head motion difference) for the 10 subjects was 0.3±3.11% (0.15±3.78%). No significant difference between intra-session head motion alterations and inter-session head motion alterations was found.

### Map-wise ALFF magnitude/reliability assessment

**Fig 2:**
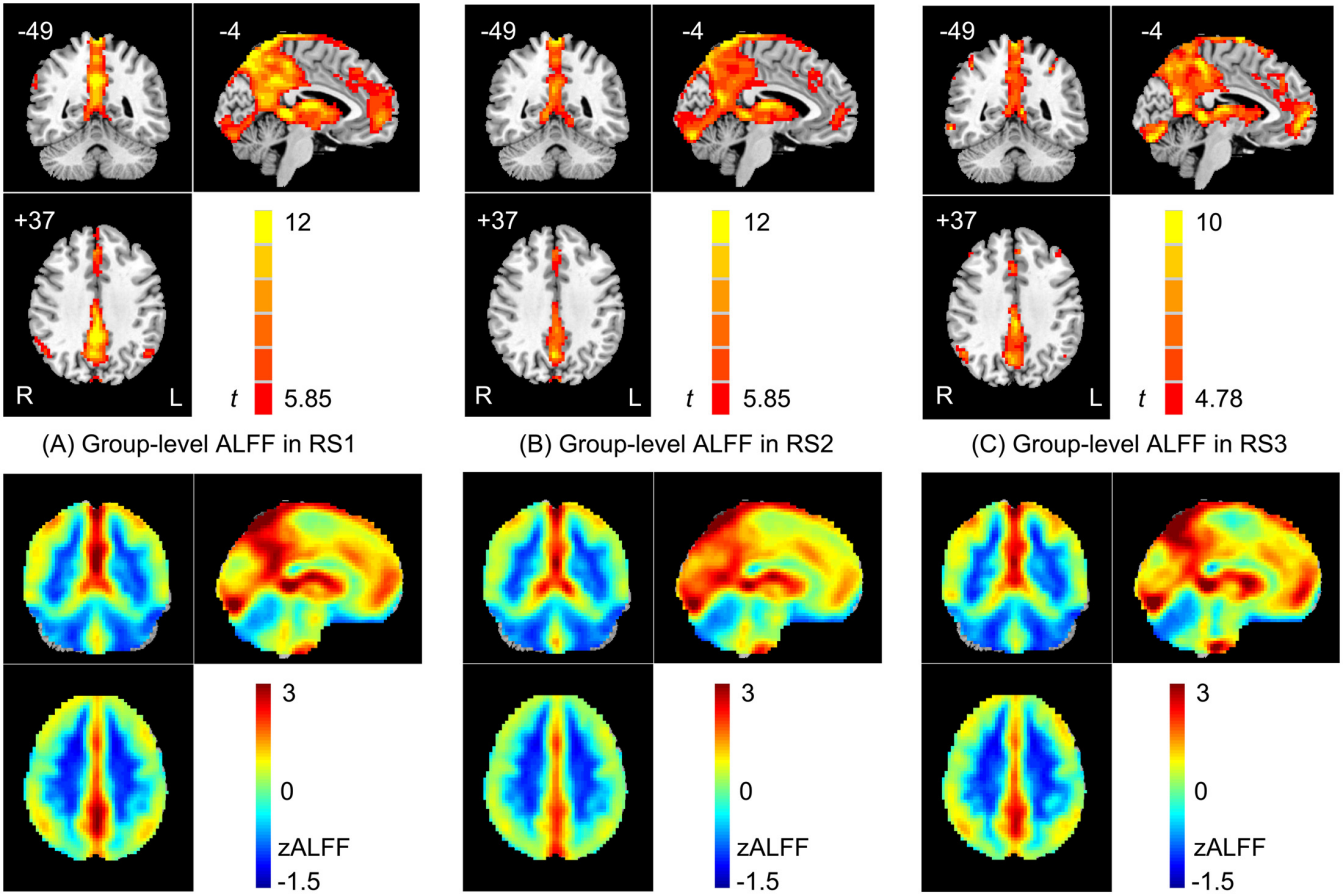
Group-level ALFF maps for the first (RS1, A), second (RS2, B) and third (RS3, C) runs. The one-sample *t*-test results shown in A and B in the upper row were generated from 21 subjects and thresholded with *t* > 5.85 (*p* < 0.00001, uncorrected) and with a cluster extension threshold of 40 voxels. The one-sample *t*-test result shown in C in the upper row was generated from 10 subjects who were scanned for a third time (*t* > 4.78, *p* < 0.001, uncorrected, cluster size > 40). Note that only the voxels with a significantly higher ALFF than the global mean ALFF are shown. In the lower row, the group-averaged zALFF without threshold for RS1, RS2 and RS3 are shown.

Significant similarity was observed among the group-level ALFF maps for the RS1, RS2 and RS3 scans (see the thresholded *t* maps for the three runs in the upper row of Fig 2). The spatial correlation among the three averaged zALFF maps was high for RS1 and RS2 (*r* = 0.964, *p* < 0.05), RS1 and RS3 (*r* = 0.969, *p* < 0.05), and RS2 and RS3 (*r* = 0.936, *p* < 0.05; see the lower row in Fig 2). The brain regions with a significantly higher ALFF magnitude than the global mean ALFF were mainly at the DMN (especially its midline structures, i.e., the posterior cingulate cortex [PCC], precuneus [PCNU], and medial prefrontal cortex [MPFC]) and thalamus, which is consistent with previous results [7,29].

### Voxel-wise ALFF magnitude/reliability assessment (21 subjects, intra-session: RS1/2)

**Fig 3:**
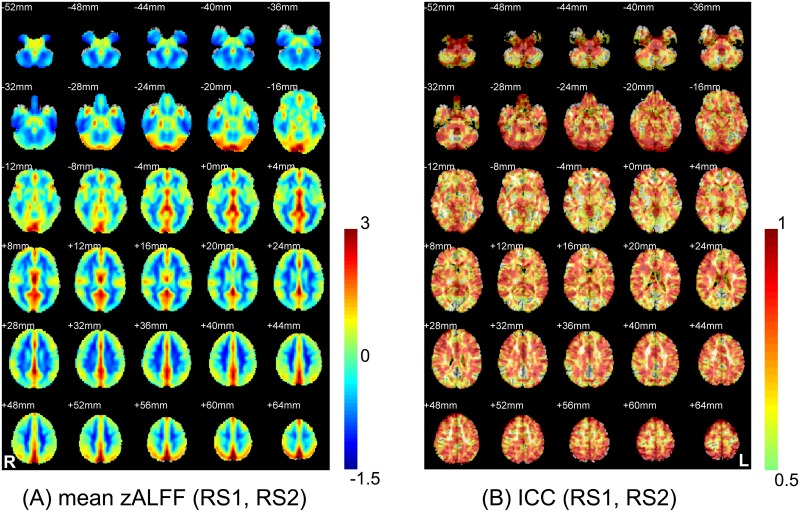
Comparison between the group-mean zALFF map and the zALFF test-retest reliability map. The two images were obtained from the 21 subjects who were scanned twice (RS1 and RS2). For a clear demonstration, the group-mean zALFF map was un-thresholded, and the zALFF intraclass correlation (ICC) map was thresholded with ICC > 0.5.

Fig 3(A) shows the mean zALFF map (without thresholding, see a thresholded mean zALFF map in S2 Fig) across all the subjects and the RS1/2. The voxel-wise intra-session test-retest reliability of ALFF (across two intra-session runs: RS1 and RS2) is shown in Fig 3(B), which was generally reliable, especially in the grey matter. However, the PCC, MPFC, and part of the thalamus, where the magnitude of the ALFF was high, exhibited poor reliability.

### Voxel-wise ALFF magnitude/reliability assessment (10 subjects, inter-session: RS1/2/3)

**Fig 4:**
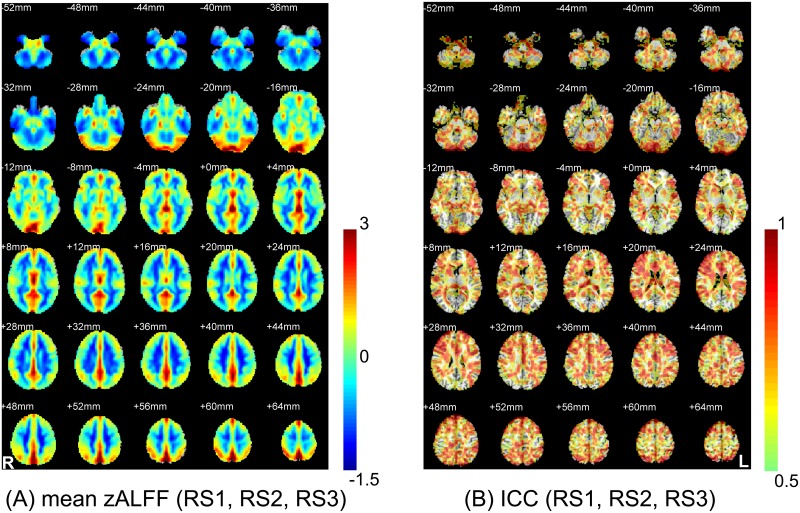
Comparison between the group-mean zALFF map and the zALFF reliability map using a three-session dataset (RS1, RS2 and RS3) from 10 subjects. The group-mean zALFF map was un-thresholded, and the zALFF intraclass correlation (ICC) map was thresholded with ICC > 0.5.

**Fig 5:**
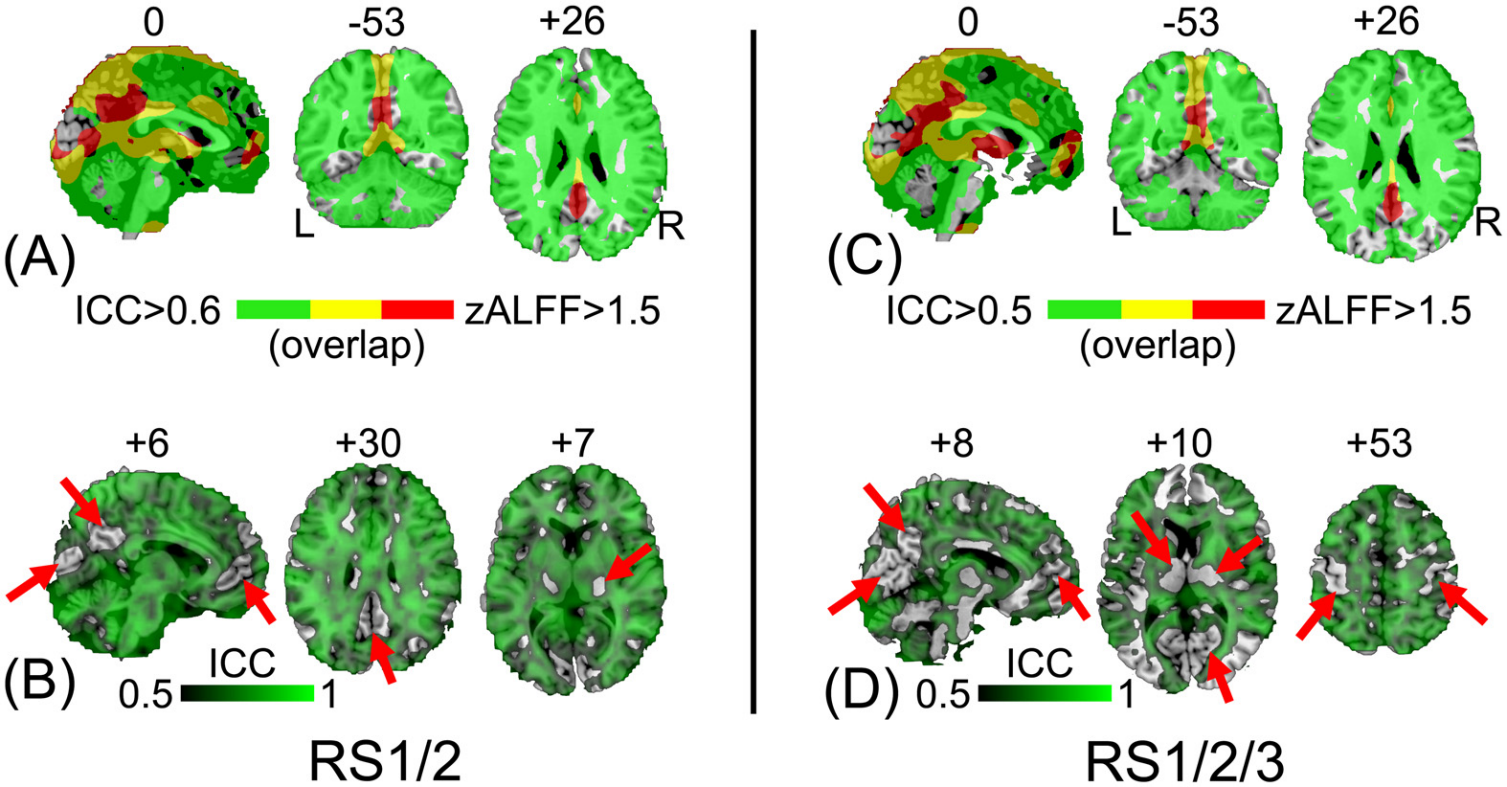
Comparisons between ALFF magnitude and reliability in an overlapping way. The left panel is the result from sessions 1 and 2 (RS1/2) using 21 subjects, and the right panel is the result from sessions 1–3 (RS1/2/3) using 10 subjects. The mean zALFF map and the ICC map were overlaid onto the CH2 template, with a green color indicating voxels with an ICC > 0.6 (a slightly higher ICC threshold is used here for intra-session reliability assessment; A) and ICC > 0.5 in the 3 run case which also included inter-session reliability (C). The red color indicates voxels with a high zALFF value (*z* > 1.5), and yellow delineates the overlap between them. For clarity, the ICC maps for RS1/2 (B) and RS1/2/3 (D) were also overlaid onto the CH2 template with a unified threshold of ICC > 0.5. Regions with no color indicate poor reliability (as shown by the red arrows).

The result was similar when we took all three runs (RS1/2/3) into consideration (Fig 4, for a thresholded version please see S2 Fig). The inter-session reliability of ALFF generally decreased, with more PVC, PMC and DMN regions having poor reliability (ICC < 0.5, Fig 4B). A detailed demonstration of the relationship between ALFF magnitude and reliability in representative slices can be found in Fig 5 (both intra- and inter-session results). An intriguing phenomenon is that the regions with low ALFF reliability exactly delineated the pattern of the DMN (Fig 5B and 5D), PVC and PMC (Fig 5D). Although the midline structure shows both high ALFF magnitude and reliability (see yellow colored areas in Fig 5B and 5D), we still noted that several key regions had mismatched ALFF magnitude/reliability (see the red colored areas in Fig 5A and 5C). Such a mismatch was preserved from intra- to intersession assessments.

### ROI-wise ALFF magnitude/reliability assessment

The ROI analysis results for ALFF magnitude/reliability are shown in Table 2. The mean ALFF magnitude in the ROIs within the DMN (including the PCC, MPFC and PCNU), thalamus, and PVC were significantly larger (single-tailed one-sample *t* test, p < 0.01) than the global mean ALFF. The rest–retest reliability of zALFF was poor (ICC < 0.5) in the PCC but was good (ICC > 0.5) in the MPFC, PCNU and thalamus. ALFF reliability was also good in the ROIs of salience network (dACC, dlPFC); however, in those ROIs, the ALFF magnitude was low (with values similar to the global mean ALFF). In the ROI within CSF, both the magnitude and the reliability of ALFF were low. These findings were consistent between RS1/2 and RS1/2/3 and robust to different subject numbers (21 or 10 subjects). However, when taking the inter-session measurement (RS3) into consideration, the reliability of the ALFF in the PMC and PVC became poor, especially for the PVC, where the reliability dropped dramatically. For an ROI within the WM, where the ALFF magnitude was low, the ALFF reliability was fair (ICC for RS1/2 is 0.48 and for RS1/2/3 is 0.57).

**Table 2:** The zALFF across subjects and the ICC value at each ROI.

ROI	zALFF (RS1/2)		ICC (RS1/2)	zALFF (RS1/2/3)		ICC (RS1/2/3)
	Mean (SD)		Mean	Mean (SD)		Mean
**PCC**	**2.50**	**(0.95)**	0.37	**2.39**	**(0.78)**	0.36
**MPFC**	**1.49**	**(0.68)**	**0.57**	**1.73**	**(0.66)**	**0.53**
**PCNU**	**1.94**	**(1.02)**	**0.66**	**2.27**	**(1.13)**	**0.68**
**dACC**	0.05	(0.32)	**0.68**	-0.03	(0.28)	**0.77**
**dlPFC**	0.22	(0.54)	**0.85**	0.37	(0.48)	**0.80**
**THA**	**1.05**	**(0.61)**	**0.79**	**0.94**	**(0.52)**	**0.63**
**PMC**	-0.12	(0.35)	**0.59**	-0.25	(0.29)	0.41
**PVC**	**1.40**	**(1.05)**	**0.54**	**1.13**	**(0.62)**	0.13
**WM**	-0.83	(0.19)	0.48	-0.89	(0.13)	**0.57**
**CSF**	-0.62	(0.18)	0.33	-0.66	(0.17)	0.39

ROI: region of interest, PCC: posterior cingulate cortex, MPFC: medial prefrontal cortex, PCNU: precuneus, dACC: dorsal anterior cingulate cortex, dlPFC: dorsolateral prefrontal cortex, THA: thalamus, PMC: primary motor cortex, PVC: primary visual cortex, WM: white matter, CSF: cerebrospinal fluid, RS1/2/3: resting-state fMRI scans 1/2/3. Note that all the ROIs were either in the midline structure or in the right side. The numbers in bold and shadow were the zALFF significantly higher than 0 (single-tailed one-sample *t* test, p < 0.01) and the ICC which was larger than 0.5.

### ALFF magnitude/reliability assessment in various frequency bands

**Fig 6:**
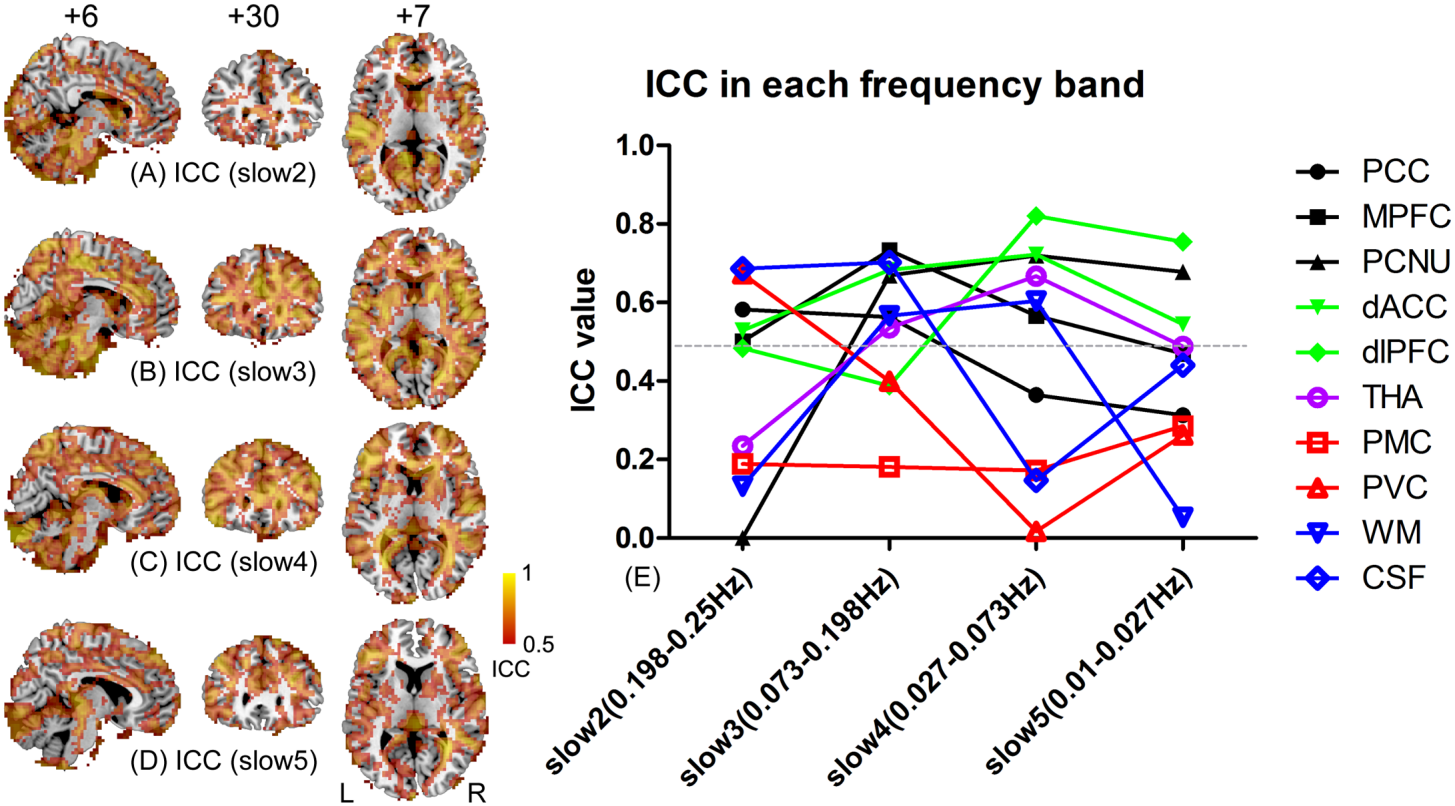
Changes in reliability of ALFF calculated at different frequency bands. The ICC maps for ALFF calculated from 10 subjects using RS1, RS2 and RS3 datasets at the frequency bands “slow 2 (0.198–0.25 Hz)” (A), “slow 3 (0.073–0.198 Hz)” (B), “slow 4 (0.027–0.073 Hz)” (C) and “slow 5 (0.01–0.027 Hz) were thresholded with ICC > 0.5. The ROI-wise ICC values were also plotted against different frequency bands (E). The 10 ROIs were selected based on previous studies or anatomical landmarks.

The frequency-specific ALFF reliability maps show large variances (Fig 6A–6D). ALFF at slow 3 produced the highest reliability with ICC globally larger than 0.5. The ALFF at slow 4 produced the second highest reliability; however, the ALFF at slow 2 and 5 had fewer brain regions with adequate reliability. The thalamic, primary visual and the default mode areas had poor ALFF reliability in all frequency bands. When plotting the ICC values against different frequency bands for the 10 pre-defined ROIs, we found that different ROIs have distinct “reliability-frequency” curves (Fig 6E). Although the PCC, MPFC and PCNU are all in the DMN, they had distinct “reliability-frequency” curves: the PCC had a more reliable ALFF in the higher frequency bands than the lower or conventional (i.e., slow 4/5) ones; the MPFC had the most reliable ALFF in slow 3, better than other frequency bands; and the PCNU had a reliable ALFF in all but the slow 2 frequency band (where the ICC was negative; thus, we set it to zero). The two key ROIs in the salience network had the most reliable ALFF measure in slow 4, as did the thalamus. The two regions in primary functional systems, i.e., the PMC and PVC, had overall poor reliability.

**Fig 7:**
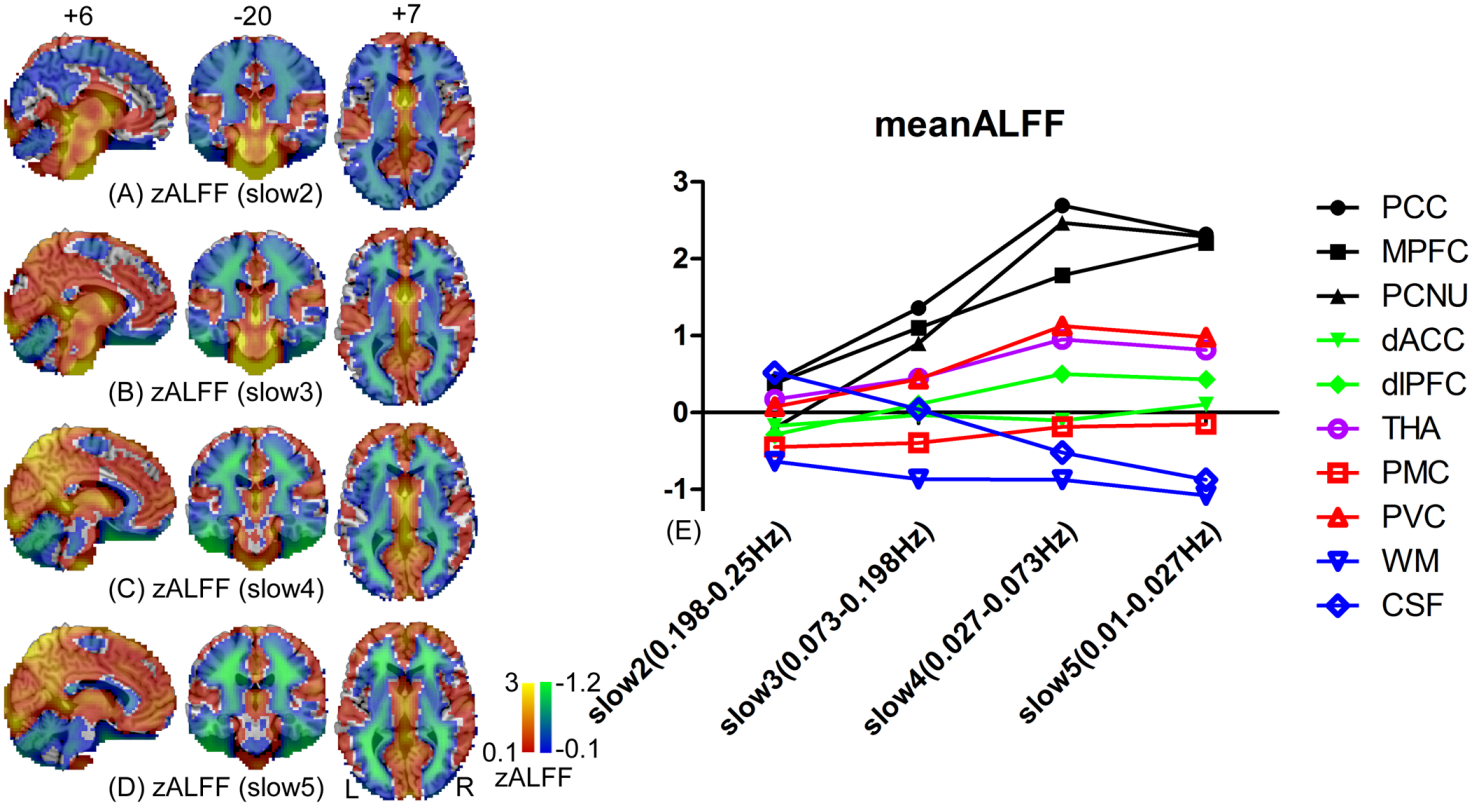
Changes in magnitude of ALFF calculated at different frequency bands. The mean zALFF maps were calculated from 10 subjects using RS1, RS2 and RS3 datasets at the frequency bands “slow 2 (0.198–0.25 Hz)” (A), “slow 3 (0.073–0.198 Hz)” (B), “slow 4 (0.027–0.073 Hz)” (C) and “slow 5 (0.01–0.027 Hz). No threshold was set to these maps. The negative zALFF is depicted by a blue-to-green color, which represents the brain regions with lower ALFF values than the global mean ALFF (i.e., the whole-brain averaged ALFF value); the positive zALFF is depicted by a red-to-yellow color, representing regions with higher ALFFs than the global mean. The ROI-wise mean zALFF values were also plotted against different frequency bands (E) for the 10 ROIs.

The mean zALFF maps in frequency bands 2–5 are shown in Fig 7 (for better representation, we did not set the threshold). Generally, the DMN regions had an increased ALFF magnitude with decreased frequency. Regions in other functional networks (the dACC, dlPFC, THA, PMC and PVC) also had increased ALFF with decreased frequency, but with the amplitude near the global mean ALFF. The non-grey-matter regions (WM and CSF) had decreased ALFF with decreased frequency bands.

### ALFF magnitude/reliability estimates against different test-retest intervals

**Fig 8:**
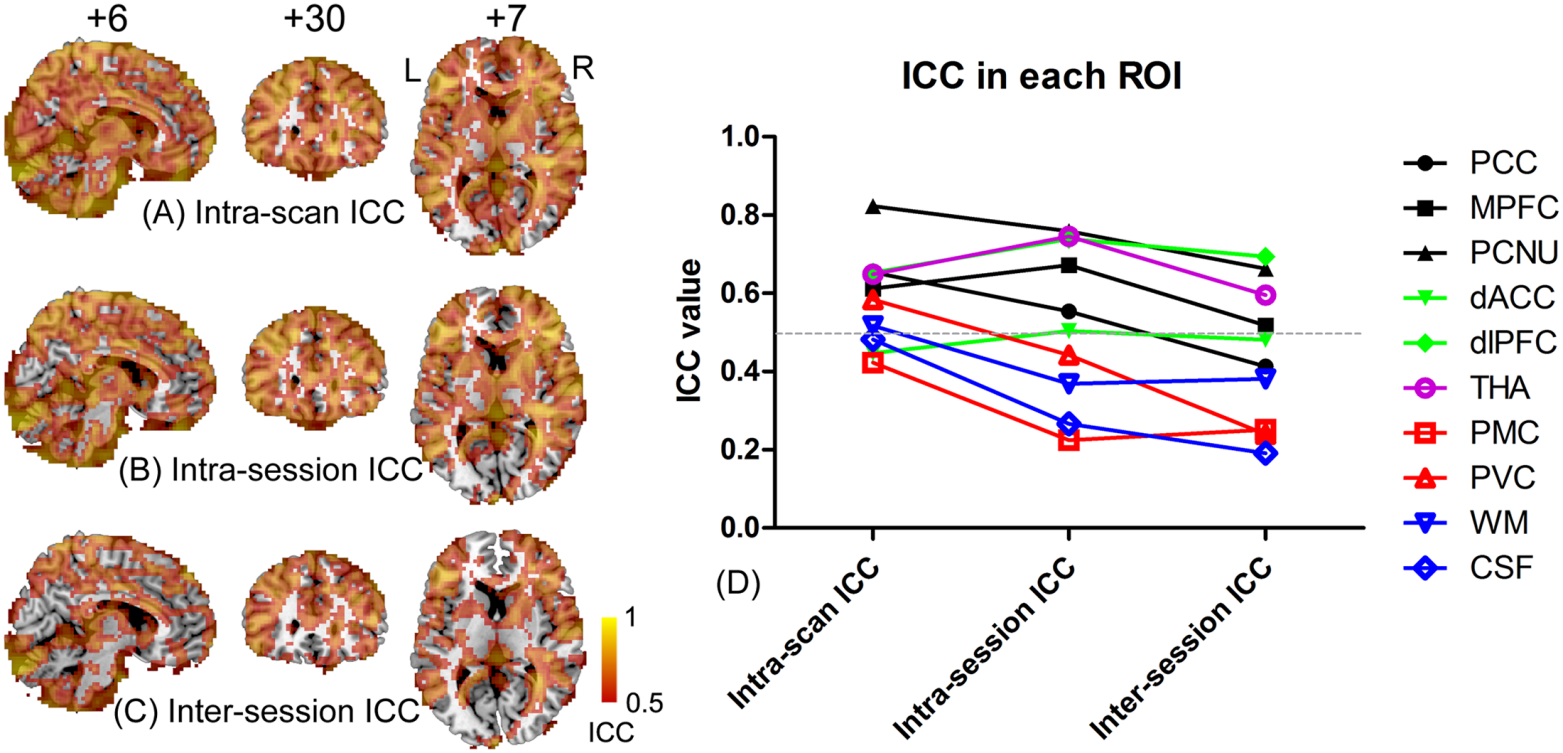
Changes in reliability of ALFF with increasing scan interval. The ICC was calculated from 10 subjects, each having three short-period data segments (156 s) for each rs-fMRI scan (RS1, RS2 and RS3). Intra-scan ICC measures reliability within each rs-fMRI scan (A); intra-session ICC (B) measures reliability within one imaging session (between RS1 and RS2); and inter-session ICC (C) measures reliability across imaging sessions (RS1, RS2 and RS3). All results were thresholded with ICC > 0.5. The ROI-wise intra-scan, intra-session and inter-session ICC were also plotted (E) for the 10 ROIs.

From short- (i.e., intra-scan) to long-term (i.e., inter-session) scanning intervals, we observed an overall reduction of ALFF reliability (Fig 8A–8C). The ALFF calculated based on segments of the data produced highly similar intra- and inter-session ICC maps to those derived based on the full-length data, but with slightly smaller ICC values. The ROI-wise ALFF reliability estimated using data segments (Fig 8D) was also similar to that based on the full-length data in Table 2. Specifically, for regions in high-order functional systems, such as PCC/MPFC/PCNU (belonging to the DMN), dACC/dlPFC (belonging to the salience network), and the thalamus (subcortical regions), the reliability was generally acceptable. For regions in primary functional systems (e.g., PMC and PVC), however, the reliability dropped quickly from acceptable to poor as the scanning interval increased (Fig 8D, red colored curves). For non-grey matter regions, the reliability was generally poor (Fig 8D, blue colored curves). The more striking finding was that the sensorimotor and visual areas had extremely low ALFF reliability, which was similar to the results based on the full-length dataset. For example, ICCs at the PVC (right calcarine, BA17, MNI coordinates [8, –67, 16]) were 0.386, 0.243 and 0.110 for the intra-scan, intra-session and inter-sessions, respectively; ICCs at the higher-order visual cortex (BA18, [27, –91, 9]) were 0.293, 0.264 and 0.208; and ICCs at the PMC (right post-central gyrus, BA4, [18, –29, 24]) were 0.378, 0.305, and 0.285.

### Validation of ALFF magnitude/reliability with an independent dataset

The mean zALFF map and the zALFF ICC map derived from the NKI-RS dataset were quite similar to the results from our data (S3 Fig) via visual inspection. These results further validated our findings.

### Reliability of ALFF based on short-TR rs-fMRI data

Using data segments from the short-TR data, we observed significantly increased intra-scan reliability over the whole brain (S4 Fig) compared with that derived from the data with a typical TR of 2s (Fig 8A). Notably, the DMN and the visual areas, with low reliability in the previous result (Fig 8A), showed adequate reliability at this time. However, regions in the intraparietal sulcus, para-Rolandic areas, middle cingulate cortex, supplementary motor area, posterior superior/middle temporal cortices, the striatum, the thalamus and the white matter areas had low reliability, and most of them also showed low reliability when 2s-TR data were used.

## Discussion

### Major findings and innovations

**Fig 9:**
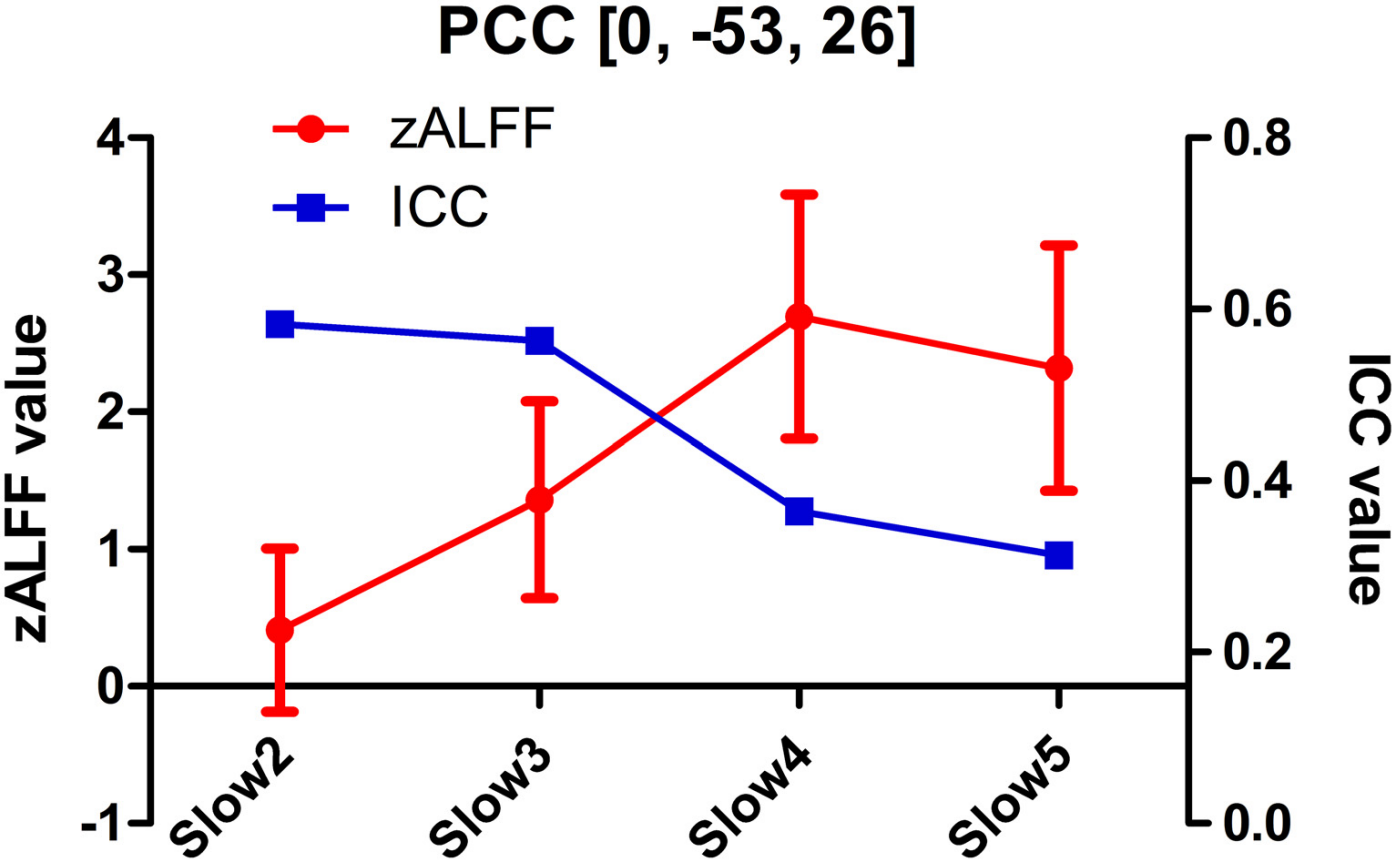
Relationship between magnitude and reliability of ALFF in the posterior cingulate cortex (PCC) across all frequency bands. The mean and STD of the zALFF across 10 subjects (RS1, RS2 and RS3) are shown in red, while the ICC of the zALFF is shown in blue. The zALFF was calculated at different frequency bands. The PCC ROI was selected centering at MNI coordinates [0, –53, 26].

In this study, we systematically investigated an interesting question: does a high ALFF magnitude necessarily indicate good reliability? The answer to this question is, as suggested by the results, “no”. Intriguingly, we found that several brain areas with high ALFF magnitudes, such as the PCC—a key node in the DMN (Figs 2, 3A, 4A, 5A and 5C), had poor test-retest reliability in terms of ALFF measurements (Figs 3B, 4B, 5B and 5D; Table 2). Moreover, as assessed in both voxel- and ROI-wise approaches, the ALFF reliability was not uniformly acceptable (Figs 3–5 and 8). Such a mismatch between the ALFF magnitude and reliability was further demonstrated with the ALFF calculated from different frequency bands (i.e., slow 2–5). That is, the higher ALFF magnitude in the PCC ROI centered at [0, –53, 26] was, the lower the ALFF test-retest reliability (Fig 9). More strikingly, the brain regions with poor ALFF reliability “perfectly” delineated the anatomical shape of the lingual gyrus (i.e., PVC) and the central sulcus (i.e., PMC) (Fig 5D). These results were further demonstrated by results obtained from ROI analyses, even though the ROI selections were blinded to the results from voxel-wise comparisons. These findings were consistent across different sample sizes and scanning intervals (Figs 5 and 8), as well as independent data sets (S3 and S5 Figs). To the best of our knowledge, this is the first study systematically comparing ALFF magnitude with reliability, and we are the first to report that several key brain regions, such as the PCC, PVC and PMC, had poor ALFF test-retest reliability.

### Potential sources inducing low ALFF reliability

The ALFF was calculated using a frequency spectrum analysis, that is, the amplitude of BOLD fluctuations. ALFF has been interpreted to reflect the strength of spontaneous neuronal activity [7], which is mainly low-frequency dominant [38]. From the spontaneous neuronal activity to the recorded BOLD signal, a great number of nuisance factors may be involved [3] that may reduce ALFF reliability. Hence, a dedicated noise reduction procedure should be utilized to estimate “cleaner” ALFF, and consequently, biomarkers can be more reliably detected in clinical neuroscience research. Without noise reduction, it is reasonable to doubt the reliability of the ALFF. Previous ALFF studies often omit noise removal processing, such as regressing out head motion parameters and the signal from non-brain tissues, which may cause unreliable results to be obtained. Our results suggest that researchers should interpret ALFF results carefully by double-checking the fMRI data preprocessing procedures. As Zuo et al. [39] and Yan et al. [37] suggested, the inclusion of nuisance signal regression, such as a global signal, white matter and CSF signals, and various head-motion parameters, may increase ALFF reliability, thus reducing the risk of reporting ALFF difference in regions with poor reliability.

The result from the short-TR datasets suggested that high-frequency artifactual signals may alias into low-frequency bands and contaminate ALFF estimation. Such artifactual signals include physiological noise such as cardiac- and respiratory-fluctuation-related signals, as well as high-frequency MR instrumental noise. This factor may, although not completely, be the cause of the low reliability of ALFF calculated from our 2s-TR dataset. The fact that short-TR data produced better ALFF reliability than 2s-TR data supports this conclusion. Future ALFF studies should take the sampling rate into consideration to improve reliability. Another reason for increasing reliability for short-TR data might be the increased SNR levels (due to the thicker slices in short-TR data). On the other hand, high-frequency signals are not only pure noise but also contribute to functional connectivity [40]. This further complicates the ALFF calculation. Band-pass filtering before ALFF calculation or calculating ALFF within the canonical low-frequency band may not always result in a reliability increase. Taking the PCC ROI for an example, “ALFF” calculated in frequency bands of slow 2 and 3 (0.073–0.25 Hz) had higher reliability than that calculated in slow 4 and 5 (0.01–0.073 Hz) (Fig 9).

The decreasing ICC with increasing scanning intervals (from intra-scan to intra-session and then to inter-session assessment) presents a good opportunity to investigate potential factors that influence ALFF reliability. Subject-related factors such as alteration in attention, mind wandering, emotional changes (increased anxiety), anticipating during scanning, and uncomfortable feelings will further reduce the reliability. The dynamics of the brain functional network may also lead to variability in ALFF estimations within a single 8 min scan. Brain areas with low ALFF reliability in Fig 8 and in S4 Fig show what may indicate such a dynamic characteristic, which will be an interesting topic for future studies. Such a dynamic characteristic also led to reduced ALFF reliability that was estimated using partial data (Fig 8B and 8C) when compared with that using full-length data (Figs 3 and 4).

Head motion, especially the FD, was proposed to significantly affect functional connectivity results [37]. Notwithstanding, we could not rule out such an effect on ALFF calculations, and we also checked whether or not it contributed to the reliability drop from intra- to inter-session assessments. No statistically significant difference was found between intra-session head motion alterations and inter-session head motion alterations, indicating that the head motion could not be the reason for lower inter-session reliability compared with intra-session or intra-scan reliability.

### Indications of mismatched ALFF magnitude and reliability

Regarding different ALFF magnitudes and reliability in different ROIs, we found four types of results: (1) both ALFF magnitude and reliability were high (e.g., PCNU); (2) both were low (e.g., CSF); (3) ALFF magnitude was high but ALFF reliability was poor (e.g., PCC, PVC), and (4) ALFF magnitude was low but reliability was good (e.g., dACC, dlPFC). Type-1 findings are ideal to report. Type 2 findings are not within our research interests because most of the regions with such results were non-grey matter areas. The mismatched ALFF magnitude and reliability was indicated by the type 3 and type 4 results. However, high (low) ALFF magnitude did not always correspond to low (high) reliability. For example, the spatial correlation between the mean zALFF and the ICC maps in Fig 4 was only 0.041 across all brain voxels and 0.031 across all grey matter voxels.

Of note, types 3 and 4 should be interpreted with caution, and they have different indications. Brain regions with type-4 results should be examined in future ALFF studies, although their ALFF values were not significantly larger than the global mean ALFF, because ALFF will be calculated reliably there. Therefore, an explicit mask with a higher ALFF magnitude than the global mean should not be used in group comparisons of ALFF [10,16]. That is, we suggest performing group difference analysis on ALFF within the whole brain or the grey matter mask, rather than first conducting within-group one-sample *t*-tests for mask generation. The latter approach has been widely used in seed-based or independent component analysis-based functional connectivity studies, the hypothesis behind which is that only the brain regions with significant functional connectivity should then be used for group comparisons. However, in ALFF studies, there is no such hypothesis. Brain regions with low ALFF magnitudes are also of great importance to researchers in the field.

Brain regions with type-3 results should be carefully checked because of the low reliability. As listed in Table 2, we used only the PCC and the PVC for example; but this type of result should not be restricted to these two regions. Fig 5 shows more brain regions with averaged zALFF > 1.5, but ICC < 0.5, such as medial frontal cortices, anterior cingulate cortices and part of the thalamus. Interestingly, according to the literature, it is quite easy to obtain type-3 results when comparing ALFF between groups, such as the group difference in the ALFF values being reported in the PCC [11,17,18], MPFC [15], both PCC and MPFC [20], thalamus [12] and PVC [9,13]. We assumed that this is due to the high ALFF magnitude and less individual variability in these regions. We suggest that in future ALFF studies, reliability or reproducibility assessment of ALFF should also be conducted in addition to comparisons of ALFF magnitudes among different groups. Therefore, we recommend collecting test-retest data or conducting a split-half reliability assessment if possible [14,21]. If practically impossible, one should separate individual rs-fMRI data into several segments and use partial data to conduct ALFF reliability evaluation, similar to that which we did for intra-scan reliability assessments and for short-TR data analysis. Attention should be paid in this case to make sure that the data segment has adequate time points (at least more than 100-s data) to accurately estimate the ALFF within 0.01–0.08 Hz.

### Similarity and differences compared with previous ALFF reliability studies

Whether the regions with poor ALFF reliability coincidently resembled the patterns of the DMN, PVC and PMC, or if this was a consistent and reliable finding should be further validated by using more datasets from different centers with different scanning parameters. However, we consider that this is a genuine phenomenon because the result was validated using the independent dataset (see S3 and S5 Figs) and is largely consistent with previous studies in terms of the gross pattern of the ICC map [29,41]. For example, Zuo et al. [29] also reported the intra- and inter-session ICC values of ALFF measures in each ROI. Although there were differences in ROI definition, the gross pattern of the ROI-wise ICC are similar between the two studies.

However, striking differences were also observed between our results and Zuo et al.’s findings [29]. Taking inter-session ICC as an example, from all peak coordinates that Zuo et al. [29] reported, we extracted our inter-session ICC values estimated across 3 data segments and 3 rs-fMRI runs (see Materials and Methods section 2.10 and Fig 8) and compared these with Zuo et al.’s report. Of the 24 peak coordinates with good-to-excellent reliability as reported by Zuo et al. [29], 14 were found to have good reliability (ICC > 0.5) by us; one was not included in our brain mask, but 9 had poor reliability (ICC < 0.5). Although this is a quite stringent method for comparing results because the peaks in different studies do not necessarily overlap, we admit that our inter-session ICC values were uniformly lower than those reported by Zuo et al. [29]. For example, the inter-session reliability at the right cuneus in Zuo et al. [29] is 0.927, i.e., excellent reliability; however, it is 0.178 based on our data.

We think that such differences were acceptable and can be explained with the following reasons. First, the state during rs-fMRI scanning was not the same between our work and that of Zuo et al. [29]. We instructed all subjects to close their eyes during scanning, but Zuo et al. instructed the subjects to open their eyes. As we cannot monitor subjects’ eye movement in the MR scanner, we cannot guarantee that all subjects keep their eyes closed all the time. This will cause variability in ALFF calculations within visual areas such as the cuneus and reduce the reliability there. Similarly, the differences in the ALFF between eyes open and eyes closed was found to lie in the sensorimotor, visual and thalamic areas [14], overlapping our findings of the regions with low ALFF reliability (e.g., PMC, PVC and thalamus). Second, the differences in MR scanners and imaging parameters could also result in such a discrepancy. Our MR scanner may have an imaging quality that is not as good as that used by Zuo et al. [29]. To assess the imaging quality, we calculated the temporal SNR for our data (data RS1, 21 subjects), for the NKI-RS data (session 1, 22 subjects), and for the short-TR data (full-length data, 46 subjects) according to the method described in [42]. The SNRs for our RS1 data, the NKI-RS session-1 data and the short-TR data are 209.5±39.5, 217.8±52.2 and 265.7±39.9, respectively. This corresponded to the overall reliability ranking: our RS1 and the NKI-RS data had comparable ICCs, but both were lower than the short-TR data (because of the thicker slices for the short-TR data), suggesting a potential contribution of the SNR to the ALFF reliability. Moreover, after regressing out the mean SNR map (generated by calculating SNR on each voxel and averaged across the 10 subjects and the three scans: RS1//2/3), the ALFF magnitude and reliability (as shown in Fig 4) was spatially positively correlated (*r* = 0.492 for all the brain voxels and *r* = 0.512 for all the grey matter voxels). This further indicates the potential contribution of the SNR on ALFF reliability, that is, if removing the SNR effect, the brain regions with high ALFF could have high ALFF reliability.

When comparing the frequency-specific ALFF with Zuo et al.’s result [29], our findings of the ALFF magnitude vs. frequency bands in WM and grey matter regions replicated their findings. That is, frequency bands of slow 2–3 contributed more to the ALFF in the WM, while slow 4–5 contributed more to the grey matter regions. In addition, our result showed that the ALFF reliability in the PCNU, dACC, dlPFC, and THA was the highest in slow 4, which was similar to Zuo et al.’s finding [29]. However, we found that the ICCs in the PVC, PCC and MPFC were higher in the higher frequency bands, which differed from Zuo et al.’s result [29]. A possible factor causing such a discrepancy is subject’ eye movement [14] during the scan as discussed above.

### Limitations

This paper aimed to investigate the relationship between the ALFF magnitude and reliability. In fALFF studies, the same problem regarding the complicated relationship between the magnitude and reliability should also be kept in mind. Another limitation is that we did not have an MR-compatible eye tracking device that can be used for real-time monitoring of eye state. This might have influenced the reliability of resting-state fMRI-based measurements such as ALFF.

## Conclusions

In this paper, we reported an interesting but problematic phenomenon: various brain regions with a high ALFF magnitude have low reliability. These regions include the midline structures of the default mode network, primary visual and motor cortices and thalamus. We suggest that reliability and consistency assessments should be incorporated in future ALFF studies, and ALFF findings should be interpreted with caution.

## Supporting Information

S1 Text ALFF magnitude and reliability calculation based on short-TR rs-fMRI data. (DOCX)

S1 Fig Comparison between ALFF magnitude and reliability using data with nuisance signals regressed out. The RS1, RS2 and RS3 data from 10 subjects were further preprocessed by removing out the averaged signals from the white matter and the cerebrospinal fluid as well as the head motion parameters. Averaged zALFF map across subjects and scans was generated and was un-thresholded for better visualization, and the zALFF intraclass correlation (ICC) map was calculated and thresholded with ICC > 0.5.
(TIF)

S2 Fig Thresholded group-mean zALFF maps. (A) Group-mean zALFF map averaged across 21 subjects and across two scans (RS1/2); (B) Group-mean zALFF map averaged across 10 subjects and across three scans (RS1/2/3). The threshold of the mean zALFF map was set to be *z* > 1.
(TIF)

S3 Fig ALFF magnitude and reliability calculated based on validation data. Comparison between the group-mean zALFF map (A) and the ALFF reliability map (B) obtained from an independent validation dataset. For validation purposes, the NKI-RS TRT dataset was employed (fcon_1000.projects.nitrc.org/indi/pro/eNKI_RS_TRT/FrontPage.html). Twenty-two subjects were involved in the production of these results. Similar data-processing procedures as those described in the main text were carried out. The mean zALFF was maintained un-thresholded, but the ICC map was thresholded with ICC > 0.5.
(TIF)

S4 Fig ICC map for short-TR rs-fMRI data. The ICC map, thresholded by ICC > 0.6, was the estimation of intra-scan reliability of the ALFF calculated at the frequency band between 0.01 Hz and 0.08 Hz.
(TIF)

S5 Fig ICC maps based on our data and that based on the validation data. ICC maps obtained from scan 1 and 2 (A), and scans 1, 2 and 3 (B). An ICC map generated from two scans using the validation data (C). All the ICC maps were thresholded with ICC > 0.5 and overlaid to the CH2 template.
(TIF)
