# Supplementary material for: Low-Frequency Fluctuations of the Resting Brain: High Magnitude Does Not Equal High Reliability
Source: PLoS One. 2015 Jun 8;10(6):e0128117. doi: 10.1371/journal.pone.0128117 (PMC4460034; doi:10.1371/journal.pone.0128117)
Supplement: S1 Text — (DOCX) [file pone.0128117.s001.docx]

**ALFF magnitude and reliability calculation based on short-TR rs-fMRI data**

A resting-state fMRI dataset of 46 subjects (age 22-32 years, 23 females) acquired with a 3.0 Tesla GE Discovery MR750 scanner in Center for Cognition and Brain Disorders (CCBD), Affiliated Hospital of Hangzhou Normal University was used for this analysis. The scanning parameters for the rs-fMRI were: EPI sequence, TR = 400 ms, TE = 15 ms, Flip angle = 30 degree, Slice number = 13, Slice thickness = 6 mm, Slice gap = 1 mm, FOV = 240×240 mm^2^, Matrix size = 64×64. The scanning parameters for the 3D whole-brain T1 image were: MPRAGE sequence, 176 sagittal slices, Slice thickness = 1mm, TR = 8100 ms, TE = 3.1 ms, Flip angle = 8 degree, FOV = 250×250 mm^2^. We also acquired a T1 FLAIR image for each subject with the same slice positions with following parameters: 13 axial slices, thickness/gap = 6/1 mm, TR/TE = 2382/25 ms, Flip angle = 90 degree, FOV = 240×240 mm^2^, Matrix size = 512×512. During the rs-fMRI scan, the subjects were instructed to keep their eyes closed and to lie still.

Each subject’s rs-fMRI data was split into three segments, each of which consisted of 156 s data (390 volumes). Data preprocessing procedure included: (1) coregistration between 3D T1 image and T1 FLAIR image using SPM; (2) 3D T1 image normalization to MNI space; (3) removal of the first 50 volumes (20-s data); (4) slice timing correction and head motion correction; (5) rs-fMRI data normalization using the deformation parameters generated from the step 1; (6) data re-sampling to 3×3×3 mm^3^; (7) spatial smoothing with an FWHM = 6 mm Gaussian kernel.

The ALFF was calculated within the frequency band of 0.01-0.08 Hz. Intra-scan ICC was calculated across the three segments. Because the reliability was estimated in an intra-scan manner rather than an inter-session manner, we set a higher ICC threshold to the final ICC maps (i.e., ICC > 0.6 means acceptable reliability).

The result was shown in the Supplementary Figure 3. Generally, only white matters and subcortical regions had low test-retest (TRT) reliability. Few default mode network-related regions had low TRT reliability. Visual corties had high TRT reliability. However, the regions near the intraparietal sulcus and the Rolandic areas, as well as the regions in the middle cingulate cortex, the supplementary motor areas, and the posterior superior/middle temporal cortices had low TRT reliability. Most of these regions also had low TRT reliability as found from the fMRI data with TR = 2s.
