# Supplementary figures and images for: Low-Frequency Fluctuations of the Resting Brain: High Magnitude Does Not Equal High Reliability
Source: PLoS One. 2015 Jun 8;10(6):e0128117. doi: 10.1371/journal.pone.0128117 (PMC4460034; doi:10.1371/journal.pone.0128117)

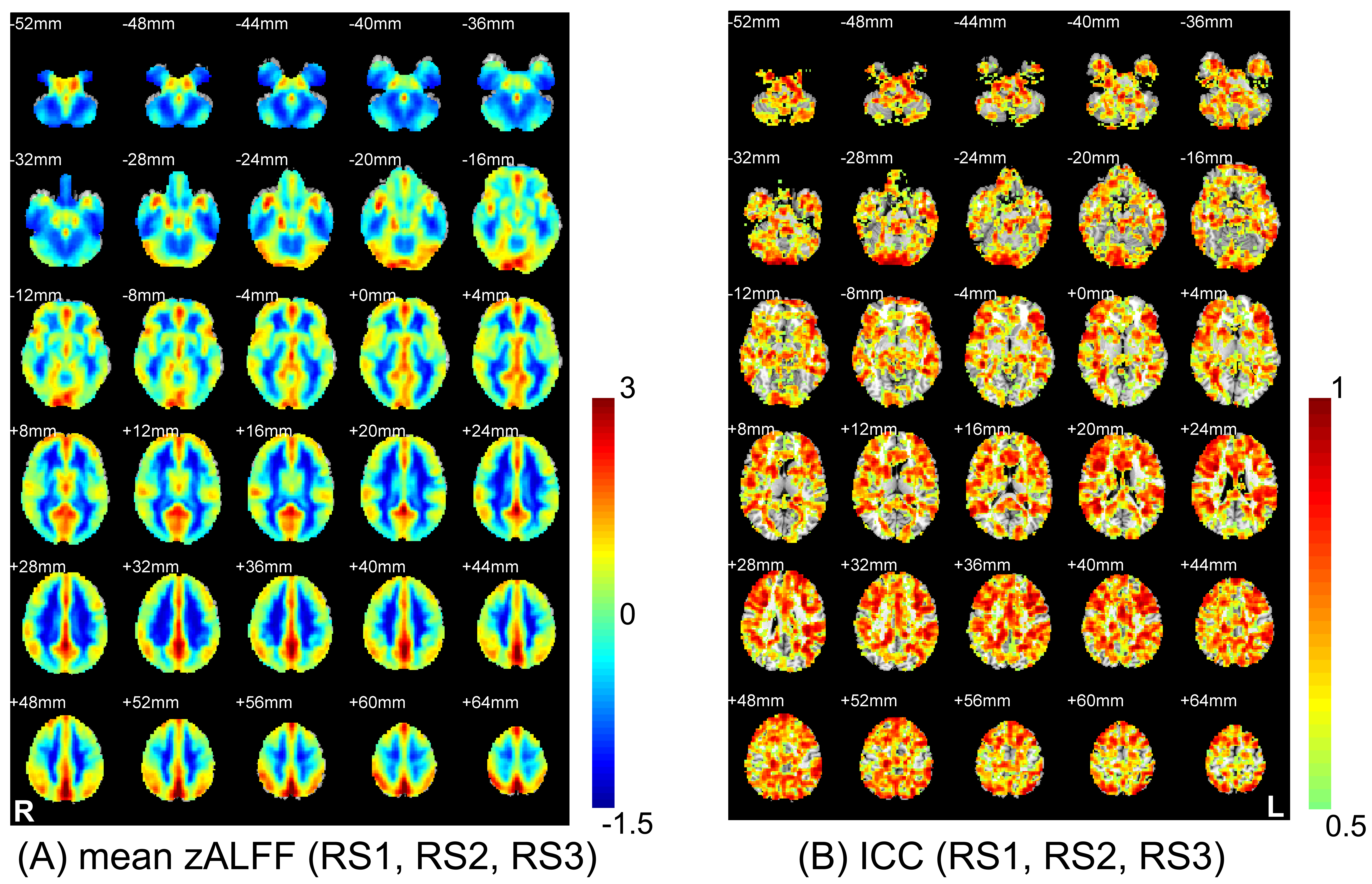

Supplement: S1 Fig — The RS1, RS2 and RS3 data from 10 subjects were further preprocessed by removing out the averaged signals from the white matter and the cerebrospinal fluid as well as the head motion parameters. Averaged zALFF map across subjects and scans was generated and was un-thresholded for better visualization, and the zALFF intraclass correlation (ICC) map was calculated and thresholded with ICC > 0.5. (TIF) [file pone.0128117.s002.tif]

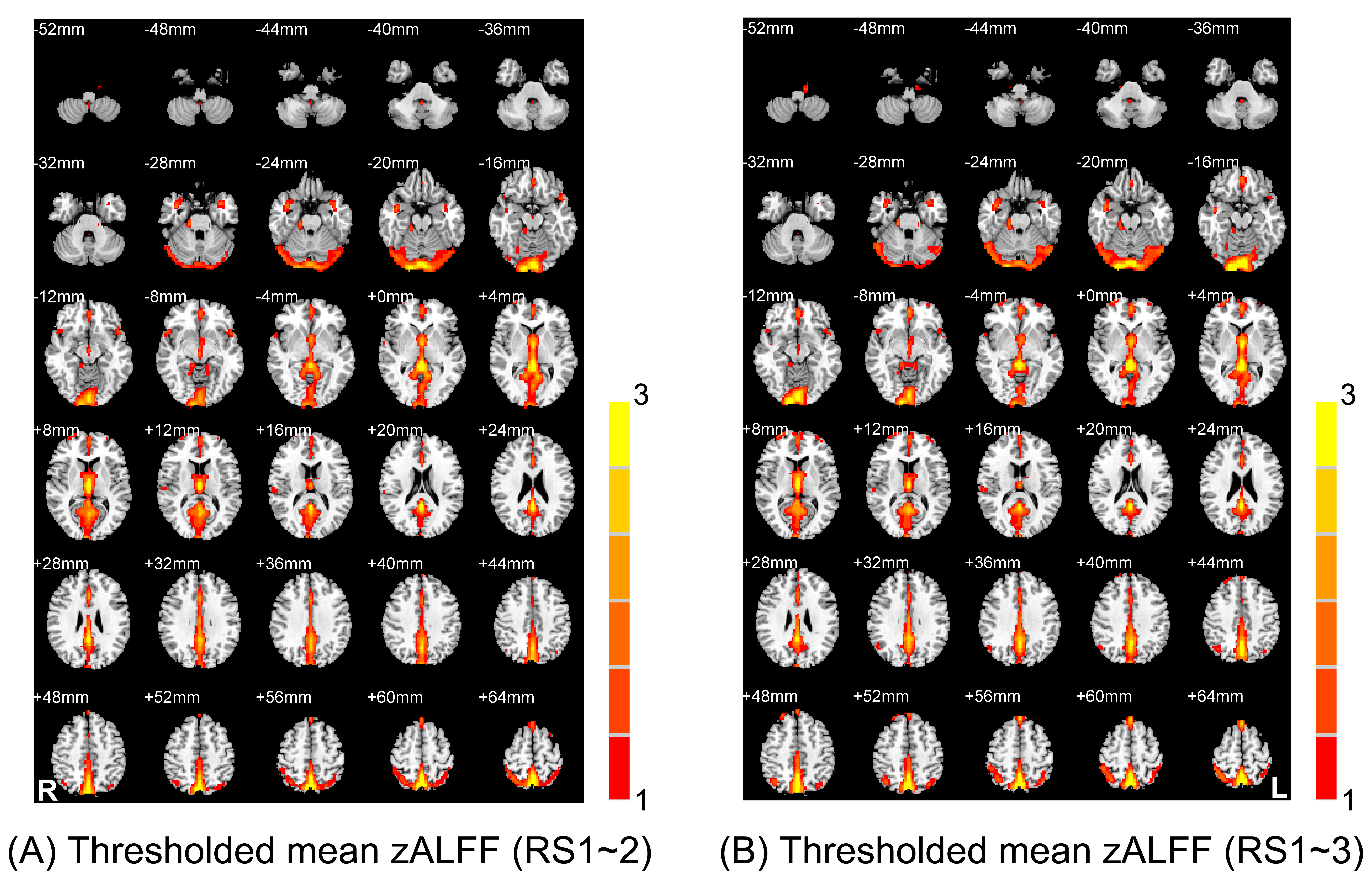

Supplement: S2 Fig — (A) Group-mean zALFF map averaged across 21 subjects and across two scans (RS1/2); (B) Group-mean zALFF map averaged across 10 subjects and across three scans (RS1/2/3). The threshold of the mean zALFF map was set to be z > 1. (TIF) [file pone.0128117.s003.tif]

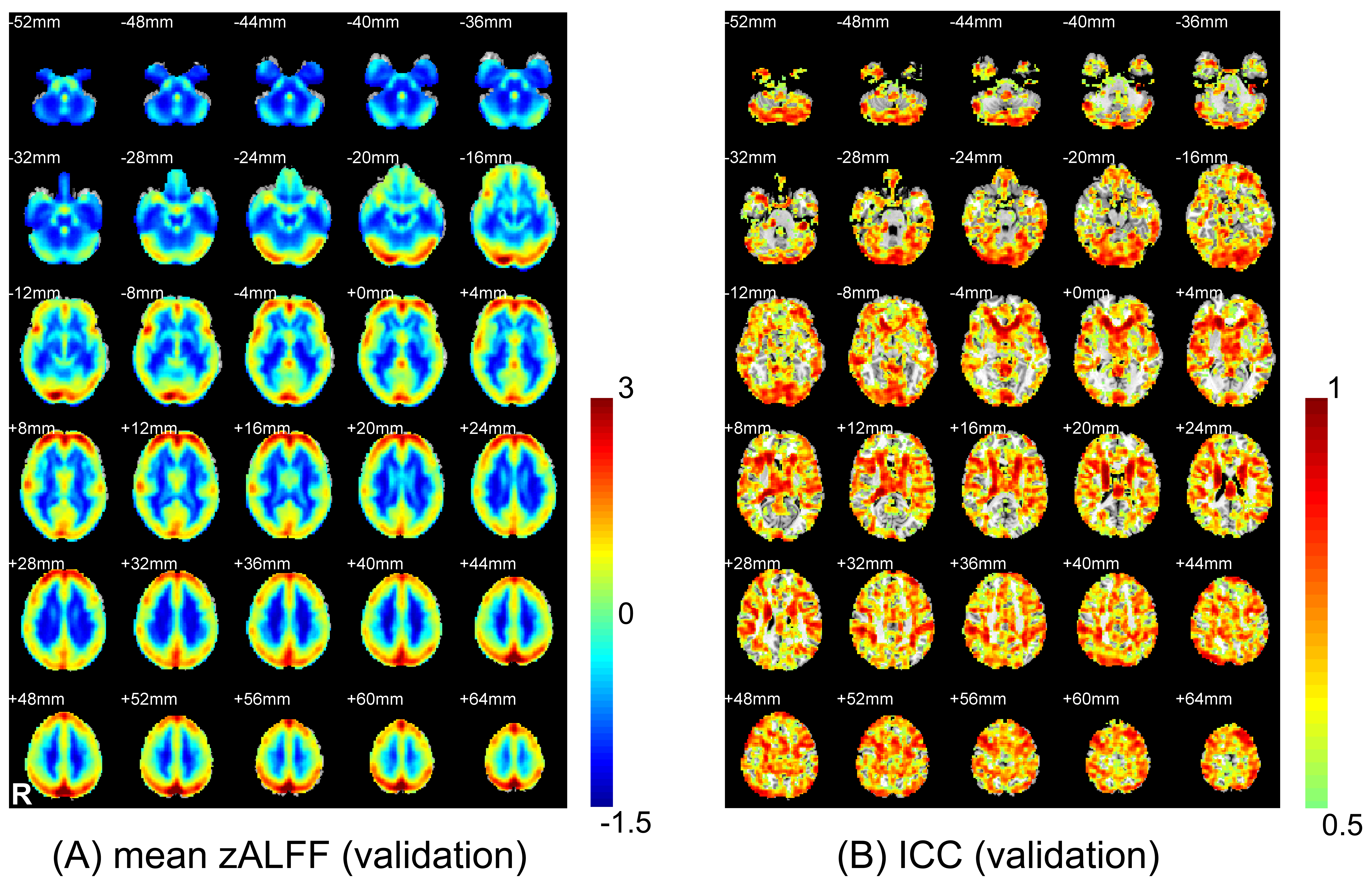

Supplement: S3 Fig — Comparison between the group-mean zALFF map (A) and the ALFF reliability map (B) obtained from an independent validation dataset. For validation purposes, the NKI-RS TRT dataset was employed (fcon_1000.projects.nitrc.org/indi/pro/eNKI_RS_TRT/FrontPage.html). Twenty-two subjects were involved in the production of these results. Similar data-processing procedures as those described in the main text were carried out. The mean zALFF was maintained un-thresholded, but the ICC map was thresholded with ICC > 0.5. (TIF) [file pone.0128117.s004.tif]

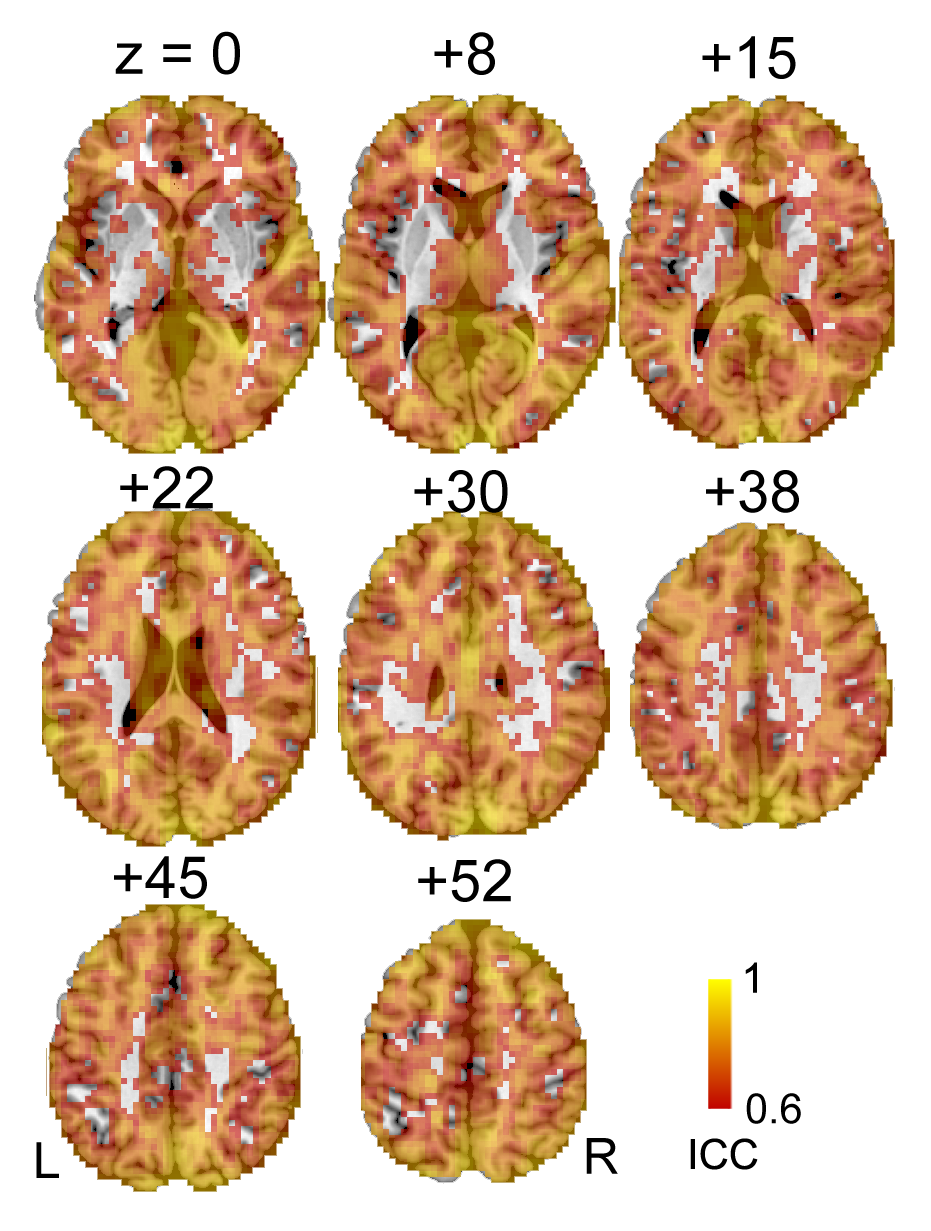

Supplement: S4 Fig — The ICC map, thresholded by ICC > 0.6, was the estimation of intra-scan reliability of the ALFF calculated at the frequency band between 0.01 Hz and 0.08 Hz. (TIF) [file pone.0128117.s005.tif]

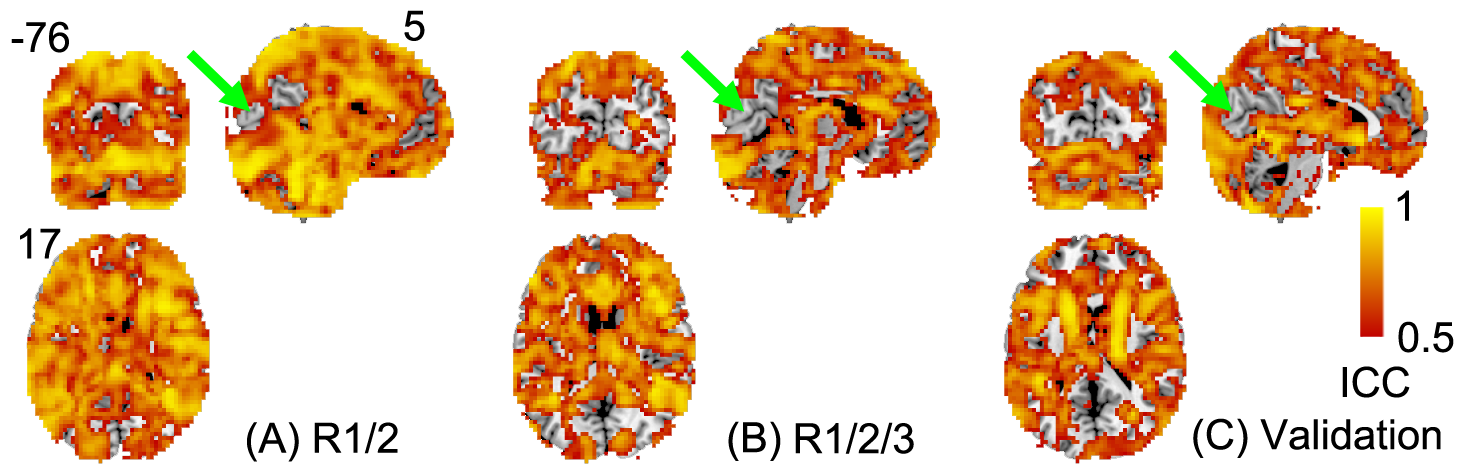

Supplement: S5 Fig — ICC maps obtained from scan 1 and 2 (A), and scans 1, 2 and 3 (B). An ICC map generated from two scans using the validation data (C). All the ICC maps were thresholded with ICC > 0.5 and overlaid to the CH2 template. (TIF) [file pone.0128117.s006.tif]
